# Current Advances in Pediatric Onset Multiple Sclerosis

**DOI:** 10.3390/biomedicines8040071

**Published:** 2020-03-28

**Authors:** Kristen S. Fisher, Fernando X. Cuascut, Victor M. Rivera, George J. Hutton

**Affiliations:** 1 Baylor College of Medicine, Texas Children’s Hospital, Houston, TX 77030, USA; kristen.fisher@bcm.edu; 2 Baylor College of Medicine, Maxine Mesinger Multiple Sclerosis Center, Houston, TX 77030, USA; fernando.cuascut@bcm.edu (F.X.C.); vrivera@bcm.edu (V.M.R.)

**Keywords:** multiple sclerosis, pediatric multiple sclerosis, neuroimmunology, demyelinating disease, pediatric neurology, child neurology

## Abstract

Multiple sclerosis (MS) is an autoimmune inflammatory disease affecting the central nervous system leading to demyelination. MS in the pediatric population is rare, but has been shown to lead to significant disability over the duration of the disease. As we have learned more about pediatric MS, there has been a development of improved diagnostic criteria leading to earlier diagnosis, earlier initiation of disease-modifying therapies (DMT), and an increasing number of DMT used in the treatment of pediatric MS. Over time, treatment with DMT has trended towards the initiation of higher efficacy treatment at time of diagnosis to help prevent further disease progression and accrual of disability over time, and there is evidence in current literature that supports this change in treatment patterns. In this review, we discuss the current knowledge in diagnosis, treatment, and clinical outcomes in pediatric MS.

## 1. Introduction

Multiple sclerosis (MS) is a chronic autoimmune disease of the central nervous system resulting in inflammation and demyelination in the brain and spinal cord. Although it is most commonly seen in adults, between 3–5% of patients have an onset of disease under the age of 18, and less than 2% of patients under 10 years of age [1,2,3,4]. Pediatric MS is rare, much less common than adult MS. The incidence of pediatric MS has been reported in ranges of 0.13 to 0.6 cases per 100,000 children per year [5]. Due to this, there have been fewer research, publications, and natural history data on pediatric MS. With the development of the International Pediatric MS Study Group (IPMSSG) in 2005, the knowledge base surrounding pediatric MS has increased. While the pathophysiology of the disease in the pediatric population is in line with that of the adult population, there are different challenges in the diagnosis, treatment, disease course, and clinical outcomes. In this review, we discuss the currently known environmental and genetic risk factors of pediatric MS, varying clinical presentations, diagnostic criteria and differential diagnoses, diagnostic evaluations, current treatment options, cognitive impairments and psychiatric comorbidity, disease course, and outcomes.

## 2. Epidemiology

In the pediatric MS population diagnosed before puberty, the number of males and females diagnosed is relatively equivalent [6,7]. In adolescents, the ratio of females to males with MS increases to 2 to 3:1, which may suggest that the onset of menarche plays some role in the pathogenesis of MS [6]. Additionally, the prevalence of MS increases after age 10 [8]. A diverse racial and ethnic population are diagnosed with pediatric MS, one study reporting 67% self-identifying as white and 20.6% as African American [9]. In this cohort, 30.2% identified as Hispanic, while other cohorts have reported up to 52% of pediatric patients with MS or CIS as Hispanic [9,10]. There has been a significant link to the role of obesity in MS and it has been shown that adolescent obesity is a risk factor for pediatric MS [11]. Not only was adolescent obesity a risk to develop MS, but one study found that in pediatric MS, obesity was present in early childhood years [12]. Obesity has been shown to promote an inflammatory state, which could contribute to not only the pathogenesis of MS, but could also play a role in the risk of relapse and long-term management. A retrospective cohort study performed comparing pediatric MS patients who were obese vs. those with normal BMI showed that obese patients had statistically significant higher relapse rates on first-line treatments, and higher relapse rates on second-line treatments [13]. Low levels of vitamin D have been associated with an increased risk of pediatric MS and with increased rates of relapse [14,15]. In the adult population it has been shown that there is an increased risk of MS in those who smoke cigarettes, and coinciding with this, children who are exposed to smoking in the home have been shown to be more likely to develop pediatric MS than a control population [16,17]. Epstein-Barr virus (EBV) may play a role in pathogenesis and risk of MS and pediatric MS, although the mechanism remains unclear at this time [15,18]. Historically, a correlation between EBV and MS was proposed due to the similarities in the epidemiology of the diseases, and studies have shown a strong correlation in support of this [19]. EBV infection can occur at any age, and is generally asymptomatic in young children. The presence of MS-mimics such as Neuromyelitis Optica Spectrum Disorder (NMOSD) and anti-myelin oligodendrocyte antibody syndrome, may contribute to a higher frequency of EBV seronegative- antibody children diagnosed with MS. One of the main genetic risk factors found in pediatric MS is HLA DRB1*1501 [20,21]. HLA DRB1*1501 is additionally seen as a genetic risk factor in adult MS, and postulated to be associated with earlier age of onset in the adult population, although there has been varying evidence in support of this, and thus at this time cannot be attributed to age of onset. The HLA class II proteins play a role in cell-mediated immunity, leading to the suspicion that it could be a genetic marker or predisposition to developing MS [22]. There are 57 previously identified single nucleotide polymorphisms that have been associated with adult-onset MS that have also been identified in a large cohort of children with demyelinating diseases and found to be associated with increased risk of pediatric-onset MS [23]. One recently published cohort identified 32% of patients with at least one relative with MS [22], with a report of incidence in a first-degree relative of 2–5% [24]. In monozygotic twins, a concordance rate of 27% is reported vs. dizygotic twins with a rate of 2.3% [24]. There have been numerous correlated factors in pediatric MS, and likely more that have yet to be determined. Many of these factors also overlap, and more studies are needed in order to determine if there are additional genetic etiologies that may lead to a predisposition to development of pediatric MS in the context of certain environmental factors.

## 3. Clinical Presentation

In a subset of patients at the initial presentation of a demyelinating event, a diagnosis of pediatric MS can be made. Younger children will often present with multifocal symptoms, but entering adolescence it becomes more common to present with single focal symptoms more similar to that of adults [25,26]. The most commonly reported symptoms in children include sensory (15–30%), motor (30%), and brainstem dysfunction (25–41%) [27]. The clinical course in 95–98% of pediatric MS patients is relapsing remitting, compared to 85–90% in adults [27,28,29]. Less than 3% of pediatric MS cases are reported as primary progressive, compared to 10–15% in the adult population [28,29,30]. In children with a progressive course, other diagnoses should be considered. Children have been reported to have a higher relapse rate compared to adult-onset MS, especially within the first few years of diagnosis [31,32]. Studies have reported 2.3–2.8 times higher relapse rate in pediatric MS, and higher rates of relapse early in the disease if without treatment or on lower efficacy treatment [32,33,34]. These findings suggest that those with pediatric MS have a more significant inflammatory component than those with adult-onset MS [32]. Due to these factors, more recent treatment has trended toward initiation with higher efficacy medications at time of diagnosis to help target this increased inflammatory state, decrease relapse rate, and prevent accrual of disability.

## 4. Diagnosis

To make the diagnosis of MS, at least one clinical event with symptoms lasting at least 24 h must be present. Dissemination in space is the development of lesions in distinct regions of the CNS, including periventricular, cortical or juxtacortical, infratentorial brain regions, and the spinal cord [35]. Dissemination in time is demonstrated by the presence of enhancing and non-enhancing lesions at any time, or by new T2 hyperintense lesions on follow up MRI [35]. In 2007, the IPMSSG created a consensus definition for pediatric MS, which was updated in 2012 following the publication of the revised 2010 McDonald criteria for the diagnosis of MS [36]. Per the IPMSSG, pediatric MS has been defined by occurrence of any of the following [37]:

1. Two or more clinically isolated syndromes (CIS) separated by greater than 30 days involving multiple areas of the CNS;
2. One CIS associated with MRI findings consistent with dissemination in space and a follow up MRI showing at least one new lesion consistent with dissemination in time;
3. One acute disseminated encephalomyelitis (ADEM) attack followed by 1 CIS more than 3 months after symptom onset with new MRI findings consistent with dissemination in space;
4. CIS with MRI findings consistent with dissemination in time and space if the patient is at least 12 years of age.

In children with acute demyelinating attacks consistent with MS, the 2010 McDonald criteria have been studied and shown to have high sensitivity, specificity, and positive predictive value for children at least 11 years of age [38]. The criteria have only a 55% positive predictive value in children under 11, and should not be applied to those with an ADEM presentation [38]. More recently, the revised 2017 McDonald criteria were compared to the prior 2010 criteria in a pediatric cohort and demonstrated improvement in accuracy (87.2% vs 66.7%) and sensitivity (84.0% vs 46.8%), but the 2017 criteria remain unvalidated in children under 12 years of age [39]. Although there is improved accuracy and sensitivity with these revised criteria, there continues to be misdiagnosis due to overlapping features with numerous mimickers of MS including multiphasic ADEM, NMOSD, vasculitis and other neuroinflammatory diseases, metabolic disorders, and leukodystrophies.

In the pediatric population, there is a wide array of alternative diagnoses to be considered when evaluating a patient for possible pediatric MS. Most commonly reported indications of an alternative etiology include progressive course at disease onset, encephalopathy, fever, negative oligoclonal bands, and significantly elevated CSF white blood cells or protein [40]. Typical lesions are ovoid in shape, and are asymmetric in hemispheric involvement. Typically, lesions are located in periventricular and juxtacortical white matter, corpus callosum, pons, cerebellum and middle cerebellar peduncle, and spinal cord (more commonly the cervical cord). MRI features that could indicate a diagnosis other than pediatric MS include symmetric bilateral lesions, large gray matter involvement at the onset, DWI abnormalities, meningeal enhancement, presence of hemorrhage, prolonged period of contrast enhancement, and presence of edema or mass effect [40]. If the MRI and clinical presentation fall within the criteria for diagnosis, in general, lumbar puncture may not be required to aid in diagnosis; however, lumbar puncture for CSF analysis should be considered in those with atypical presentations. As there are limitations in current diagnostic criteria, evaluation for other mimics of pediatric MS, should be assessed at time of presentation with Aquaporin-4 and anti-MOG antibodies. Anti-MOG antibody can also be present in monophasic and multiphasic ADEM, which differ from MS and NMOSD in prognosis and management. Other studies to be performed at time of presentation may include (if indicated per clinical judgement) the C-reactive protein, erythrocyte sedimentation rate, anti-nuclear antibody, angiotensin-converting enzyme level, folate, vitamin B12, and thyroid-stimulating hormone. Those with a progressive course should be evaluated for mitochondrial, metabolic, and neurodegenerative disorders including leukodystrophies. Other considerations include other autoimmune conditions, including systemic lupus erythematosus, neurosarcoidosis, or Sjogren syndrome.

Clinically Isolated Syndrome (CIS) in pediatrics has been defined by the IPMSSG as a monofocal or polyfocal CNS event of presumed inflammatory/demyelinating cause in a child without encephalopathy and no prior history of CNS demyelination. The MRI should not meet the criteria for MS diagnosis, with both dissemination in time and space absent. Optic neuritis is the most common CIS presentation in pediatrics, followed by transverse myelitis and brainstem syndromes [41]. In a cohort of 770 patients with pediatric CIS who were followed for 10 years to assess the risk of conversion to MS, female gender and multifocal symptoms at onset were risk factors for the occurrence of a second attack [42]. In pediatric optic neuritis, rates of conversion to MS range from 13.8–32% [43,44]. There has been a higher risk of conversion to MS reported in those with abnormal brain MRI at the onset of optic neuritis, bilateral optic neuritis, and those with recurrent optic neuritis [43,44,45]. Transverse myelitis, similarly, is typically a monophasic disorder. Reported risk factors for relapse of pediatric transverse myelitis include female gender and abnormal brain MRI, which is consistent with that reported in adult studies [46].

Acute disseminated encephalomyelitis (ADEM) is most commonly a monophasic demyelinating disease preceded by viral infection or vaccination. Patients typically present with multifocal symptoms and encephalopathy, and seizures can also be present. MRI findings play a large role in the differentiation of ADEM from MS: diffuse bilateral lesions with ill-defined borders are more commonly seen in ADEM [47]. Susceptibility weighted imaging has been used in the identification of multiple sclerosis, but also has been looked at as a possible tool in helping differentiate ADEM and MS [48]. In patients with multiphasic ADEM, anti-myelin oligodendrocyte glycoprotein (MOG) antibodies can be evaluated to help differentiate from MS. Anti-MOG associated disorders can present as optic neuritis, monophasic ADEM or a neuromyelitis optica spectrum disorder (NMOSD). While some patients with anti-MOG associated disorders will respond to an initial course of steroids, some will require longer-term immunomodulation [49].

Most commonly, patients with NMOSD present with transverse myelitis and optic neuritis. MRI findings are used in differentiating pediatric MS from NMOSD. Patients with NMOSD can have brain MRI abnormalities, most commonly reported in the diencephalic region, dorsal medulla (area postrema), and peri-ependymal circumventricular areas. Typical characteristics of optic nerve lesions in NMOSD include involvement of the optic chiasm and lesions extending greater than half the length of the optic nerve [50]. In NMOSD, spinal cord lesions extend at least three vertebral segments, which is seen in 10% of patients with pediatric MS [47]. Additionally, it has been seen that contrast enhancement in spinal cord lesions in NMO display a rim-enhancement as compared to MS where more uniform enhancement is seen [51]. Due to selective involvement in the area postrema, 38% of pediatric NMO patients present with vomiting [52]. CSF can display significant pleocytosis with neutrophilic or lymphocytic predominance, and oligoclonal bands are less frequently seen than in MS [53,54].

## 5. Diagnostic Evaluations

MRI is an important tool in pediatric MS and aids in the diagnosis. MRI findings are used for the assessment of dissemination in time and space. Spinal cord imaging typically shows cervical cord lesions, short segment lesions (< 3 vertebral segments in length) that involve only a portion of the diameter of the cord [55]. While MRI brain and spine are the most valuable test in supporting the diagnosis of MS, the MRI spine may be less useful in the diagnosis of pediatric MS, with one study only showing 10% of patients meeting the criteria of dissemination in time and space based on the addition of MRI spine [56]. Based on the initial MRI, pediatric MS patients show a higher number of T2 lesions than adults and more frequently have cerebellar and brainstem involvement [57]. Additionally, pediatric patients have lesions that are larger and more ill-defined [58]. The presence of at least one periventricular white matter lesion and at least one T1 hypointensity on initial MRI can predict progression to MS at the time of presentation [59].

Cerebrospinal fluid (CSF) pleocytosis is present in 53–66% of patients, with cell count reported as high as 61 [60,61]. A lymphocytic predominance is more commonly seen, but in children under 11 years of age, an elevated neutrophil count may be seen [8]. The presence of oligoclonal bands in the CSF is significant for ongoing neuroinflammation and has been reported in numerous CNS inflammatory conditions, including pediatric and adult MS. The presence of oligoclonal IgG bands in the CSF has been reported in 64% to 92% of pediatric MS patients, and is seen to be less frequent in younger children [60]. Oligoclonal bands in the CSF can be used to aid in the diagnosis of pediatric MS. In children aged 12–17 years old with clinical suspicion of MS, the presence of oligoclonal bands strongly supports a diagnosis [62]. In patients with a pediatric radiologically isolated syndrome, the presence of oligoclonal bands increases the specificity of MRI criteria, and can help in predicting conversion to MS [63]. It is important to note that while oligoclonal bands can be used to make the diagnosis of pediatric MS, other conditions that are classified as mimickers of pediatric MS can also have a presence of oligoclonal bands, thus, this presence does not eliminate other diagnoses.

## 6. Treatment Options

The primary goal of treatment with disease modifying therapy is to achieve a state of no evidence of clinical or radiographic disease activity. No evidence of disease activity (NEDA) is characterized by the absence of clinical relapses, no progression of clinical disability, no new or enlarging T2 lesions on MRI, and no contrast-enhancing lesions on MRI [64]. There are varying approaches to disease modifying therapy, including an escalation or “step-up” vs. an induction or “step-down” approach [6]. The first approach involves starting with what is considered to be first-line therapy, and escalating treatment if the patient were to have evidence of clinical relapse or interval development of new demyelinating lesions on MRI despite patient compliance with the medication and adequate duration of treatment [6]. The second involves using the more efficacious treatments first to induce a state of no evidence of disease activity. At this time, fingolimod is the only FDA approved treatment for pediatric MS, but other treatments, including interferons, glatiramer acetate, dimethyl fumarate, teriflunomide, natalizumab, rituximab, and cyclophosphamide, have been used and reports have shown the benefits of these treatments. Currently, consensus statements suggest first-line therapies as interferons or glatiramer acetate, but more recent studies have led to a discussion of the need for revision of these guidelines in light of studies showing that a large number of patients on injectable therapies require escalation of therapy [65,66]. Additionally, there is no standard definition of treatment failure across treatment centers, and with this, no guidelines for the transition of medications. The IPMSSG has proposed definitions for breakthrough disease, including an increase or no reduction in relapse rate, development of new T2 or contrast-enhancing lesions on MRI, or two or greater clinical or MRI relapses within 12 months [67]. Some children achieve a state of NEDA on first-line medications, but some require a transition of medications due to the breakthrough of disease, while other patients may change medications due to poor tolerance or non-compliance.

Interferons in injectable formulations along with glatiramer acetate are commonly used first-line treatments in pediatric MS as has been displayed with observational studies [68]. Interferons are well tolerated, with 25–35% of children reporting flu-like symptoms [69,70]. To mitigate adverse effects, the dose can be titrated over 4 weeks until reaching full dose, and pretreatment with analgesics is recommended. Regulatory laboratory monitoring is required as interferon beta can result in elevation of liver transaminases, thyroid function abnormalities and decreased peripheral blood cell counts [69,70,71,72]. Interferons should be used with caution in children with a known history of depression as there have been reported mood side effects [6]. In 44 pediatric MS patients treated with interferon-beta-1b, no serious adverse events were reported [70]. In a retrospective study named REPLAY, adult doses of interferon beta-1a were tolerated without adverse reactions in pediatric MS [69].

Glatiramer acetate is a synthetic amino acid polymer that resembles myelin basic protein, which is delivered via subcutaneous injection. Retrospective studies have shown reduction of annualized relapse rate (ARR) similar to those of the adult trials ranging from 0.2–0.25 [73,74]. In the pediatric population, full adult dosing is used and generally well-tolerated [75]. The most commonly reported side effect are injection site reactions. Rarely, an immediate post-injection systemic reaction occurs with flushing, chest pain, palpitations, and shortness of breath, but this usually self-resolves. Regular laboratory monitoring is not required. As interferons and glatiramer acetate are generally well tolerated and shown to decrease relapse rates in retrospective studies, they were commonly used as first line treatments until the more recent development and introduction of newer treatment options.

Fingolimod, a once-daily oral medication, was approved by the FDA in 2018 as a first-line treatment in pediatric MS based on the results of the PARADIGMS trial. Fingolimod binds to sphingosine-1-phosphate receptors, sequestering lymphocytes in the lymph node and prevents activated lymphocytes from crossing into the CNS. In pediatric MS, an 82% reduction in annualized relapse rate in comparison to those treated with interferon beta-1a was demonstrated [76]. Additionally, over a two-year interval at all time points, patients treated with fingolimod had lower EDSS scores compared to those treated with interferon beta-1a [77]. While the PARADIGMS trial showed improved efficacy of treatment and improved EDSS at follow up with treatment with fingolimod compared to interferon beta-1a, it additionally has increased risks with treatment. The serious adverse events in the pediatric population included leukopenia and seizures. It is required that patients be monitored for bradycardia for 6 h with the first dose. Patients should be monitored for macular edema with annual ophthalmology exams, although there was only one report of macular edema in the PARADIGMS trial [76]. Routine laboratory monitoring of liver transaminases and lymphocyte counts are recommended. Additionally, there is a risk for progressive multifocal leukoencephalopathy (PML), which has been more commonly reported in patients on fingolimod for longer duration and with positive John Cunningham Virus (JCV) antibodies. PML is an opportunistic infection of the CNS that is potentially fatal and is caused by the reactivation of latent JCV.

Dimethyl fumarate is a twice-daily oral medication that was approved by the FDA in 2013 for the treatment of MS in adults. The specific mechanism of action is unknown, but it is shown to affect cytokines and lower lymphocyte counts. Phase 3 studies in adults have shown dimethyl fumarate significantly reduces relapse rates and the development of new T2 hyperintense lesions on MRI. Common side effects include flushing, which can be abated with pretreatment with aspirin. There have been cases of PML in patients on dimethyl fumarate, all occurring in patients with lymphocyte counts under 800, thus, routine laboratory monitoring is recommended. An open-label study, FOCUS, was performed to evaluate the effect of dimethyl fumarate on MRI activity in the pediatric population, and showed a reduction in the development of new T2 hyperintense lesions [78]. While there are data showing a reduction in the breakthrough disease on MRI, there are yet to be data on the reduction of clinical relapse in the pediatric MS population. CONNECT is an open-label randomized controlled study comparing dimethyl fumarate versus interferon beta-1a in the pediatric population that is currently ongoing [79].

Teriflunomide is a once-daily oral pill that reduces the activation and proliferation of lymphocytes by inhibiting pyrimidine synthesis. In studies in the adult MS population, teriflunomide has been shown to significantly reduce the relapse rate, disability progression, and new activity on MRI in comparison to placebo [80]. Common side effects include hair thinning, nausea, diarrhea, and elevated liver transaminases. There is a significant risk of teratogenicity, and if pregnancy is desired. a period of washout with cholestyramine should be performed. Teriflunomide is not commonly prescribed in the pediatric population. TERIKIDS, a randomized, double-blind, placebo-controlled trial, is currently ongoing, evaluating efficacy and safety of teriflunomide in pediatric MS [79].

Natalizumab is a humanized monoclonal antibody that has been shown to decrease clinical relapse by 68% and decrease the development of new T2 hyperintense lesions by 83% compared to placebo in adults, and small open-label studies in pediatric MS population have shown good efficacy and tolerability [81,82,83,84]. In a study of 55 pediatric patients, only three relapses occurred, all within 6 months of initiation of natalizumab [82]. At one year follow up, 83% were free of new T2 lesions on MRI and 74% at two years follow up [82]. No serious adverse effects were reported in this study group. Additionally, a recently published cohort of 20 treatment naïve pediatric MS patients showed that over a treatment period of 24 months with natalizumab, patients had a significant reduction in mean EDSS overall, and NEDA-3 plus status (no evidence of relapse, no disease progression, no new MRI activity, and no cognitive decline) was maintained in 80% of patients, demonstrating natalizumab as a highly effective treatment in pediatric MS [85]. JCV antibodies should be monitored at least every 6 months given the elevated risk of PML in those who are JCV antibody positive, and if seroconversion were to occur, then transitioning to alternative therapy should be considered. Additional risk factors in the setting of positive JCV status include prolonged duration of natalizumab use and prior immunosuppression.

Rituximab is a monoclonal antibody that depletes CD20+ B cells that has been shown in both pediatric and adult MS populations to reduce both clinical relapses and MRI lesions [86,87]. In a case series of 14 pediatric MS patients treated with rituximab, no patients had subsequent relapses [88]. The most common adverse effects include hypogammaglobulinemia and infusion reactions [87,89]. The risk of PML is present with rituximab; however, in pediatric MS a larger population and longer follow up is needed to better understand this risk [89].

Cyclophosphamide, an alkylating agent, has been shown to be effective in reducing the relapse rate in pediatric MS patients with aggressive disease [90]. It is given as a monthly infusion, and affects cytokine expression, in addition to T-cell and B-cell function [80]. While it is effective, there are significant risks of secondary malignancies, infection, and sterility [90]. Additional side effects include nausea, vomiting, alopecia, osteoporosis, and amenorrhea [80].

## 7. Cognitive Impairment, Fatigue, and Psychiatric Comorbidities

Cognitive function is affected in approximately 30% of pediatric MS patients. Children present with differing deficits than those with adult MS, as children can show greater deficits in vocabulary and language-based cognition [91]. Other commonly reported impairments in those with longer disease duration include attention, processing speed, visual-motor skills, executive functions, and memory [92]. Cohorts of pediatric MS patients who have undergone neuropsychology evaluation have found a strong association between cognitive impairment and EDSS score, number of relapses, and disease duration [93]. Studies have looked at cognitive functioning at the time of diagnosis and in follow up. One recent study compared initial neuropsychological evaluations in 19 patients who were either treatment naïve or on solely interferon beta, all of which had follow up assessments performed. Six patients were escalated to a higher efficacy treatment (three to natalizumab and three fingolimod), and the remainder did not require escalation of treatment (10 on interferon beta-1a, two on glatiramer acetate, and one on dimethyl fumarate). While cognitive impairment was seen early in the disease at initial evaluation, those patients who did not have an escalation of treatment had a higher degree of impairment at time of follow up in comparison to those who had an escalation of therapy [94]. Studies additionally have shown that those with pediatric MS are at a higher risk of cognitive disability than adult MS patients [95,96]. Cognitive impairment is more significant in the pediatric MS population, and findings show that those who have been escalated to a higher efficacy treatment to have a lower degree of cognitive impairment; thus, this argues towards an induction or “step-down” treatment approach to help protect from development or further cognitive decline, although larger studies are warranted to confirm this hypothesis.

There are additional features of pediatric MS that can impact everyday functioning. Fatigue is a common complaint in the MS population and can significantly affect daily functioning and can also be a contributing factor in cognitive functioning. In one study comparing pediatric MS patients with healthy controls, there was no significant difference between the two groups in regard to self-reported fatigue, although an additional study reported that at least half of pediatric MS patients report at least mild fatigue [97,98]. Importantly in this study, self- and parent-reported fatigue were associated with higher scores on the Children’s Depression Inventory [97]. Additional studies have shown signs of depression are higher in the pediatric MS population [99,100]. In combination, depression or anxiety disorders are present in approximately half of pediatric MS patients. The most commonly reported psychiatric diagnoses are anxiety disorders, attention deficit hyperactivity disorder, and mood disorders [101]. When looking at overall quality of life, one study reported that approximately half of pediatric MS patients reported difficulties in school and emotional functioning [99]. Additional studies have shown that in addition to fatigue and depression, increased EDSS can contribute to decrease in health-related quality of life [102].

## 8. Clinical Outcomes

Despite elevated relapse rates, the pediatric population tends to have a complete recovery from relapse within 12 months with little accrual of disability during childhood years [36]. Generally, as children do not accumulate disability, they will not develop secondary progressive MS in childhood years, and it generally will take the pediatric MS population approximately 10 years longer than the adult MS population to convert to secondary progression [28,103]. Despite this, the time of progression from mild to severe disability is the same in adults and children [28]. Given this, those with pediatric MS will reach disability milestones at a younger age than those with adult-onset MS [30,103,104]. An increased risk of disability in pediatric MS was associated with a progressive course at onset and an increased number of relapses in the first five years, while a reduced risk was present in those that had complete remission from the initial event [103]. These findings further support the importance of early recognition and treatment in pediatric MS.

Patients with pediatric MS have a smaller brain volume than expected for their age [105], and this carries into adulthood. In comparing the adult brain volume of pediatric MS patients with age-matched adult MS patients, those with the pediatric-onset disease have reduced brain and deep grey matter volume, particularly thalamic volume [106].

## 9. Conclusions

Pediatric MS is a rare disorder, but the long-term clinical implications in those who are diagnosed lead to significant cognitive and physical disability in adulthood, even with current first-line treatments. The ultimate aim of the treatment of pediatric MS is to reach a state of NEDA. More recently, the goals have been aimed towards NEDA-4, which is a state of no clinical relapse, no disease progression, no new MRI activity, no cognitive decline, and no evidence of brain atrophy present [107,108]. Given the elevated annualized relapse rate, increased rate of cognitive disability, and younger age at reaching disability milestones in pediatric MS, to achieve NEDA-4, the treatment paradigm may need to shift towards that of treatment with higher efficacy medications.
